# NOX2ko Mice Show Largely Increased Expression of a Mutated NOX2 mRNA Encoding an Inactive NOX2 Protein

**DOI:** 10.3390/antiox9111043

**Published:** 2020-10-26

**Authors:** Monika Göllner, Irmgard Ihrig-Biedert, Victoria Petermann, Sabrina Saurin, Matthias Oelze, Swenja Kröller-Schön, Ksenija Vujacic-Mirski, Marin Kuntic, Andrea Pautz, Andreas Daiber, Hartmut Kleinert

**Affiliations:** 1 Department of Pharmacology, University Medical Center of the Johannes Gutenberg University Mainz, Langenbeckstr. 1, 55131 Mainz, Germany; mgoelln@uni-mainz.de (M.G.); ihrig@uni-mainz.de (I.I.-B.); Victoria-Petermann@web.de (V.P.); ssaurin@students.uni-mainz.de (S.S.); pautz@uni-mainz.de (A.P.); 2 Laboratory of Molecular Cardiology, Department of Cardiology 1, University Medical Center of the Johannes Gutenberg University Mainz, Langenbeckstr. 1, 55131 Mainz, Germany; Matthias.Oelze@unimedizin-mainz.de (M.O.); swenja.kroeller-schoen@unimedizin-mainz.de (S.K.-S.); kvujacic@students.uni-mainz.de (K.V.-M.); mkuntic@students.uni-mainz.de (M.K.)

**Keywords:** oxidative stress related disease, nicotinamide adenine dinucleotide phosphate (NADPH) oxidase (NOX2) knockout mice, truncated and inactive mutant, next generation sequencing (NGS)

## Abstract

Background: The superoxide-generating enzyme nicotinamide adenine dinucleotide phosphate (NADPH) oxidase (NOX2 or gp91phox, the phagocytic isoform) was reported as a major source of oxidative stress in various human diseases. Genetic deletion is widely used to study the impact of NOX2-derived reactive oxygen species (ROS) on disease development and progression in various animal models. Here, we investigate why NOX2 knockout mice show no NOX2 activity but express NOX2 mRNA and protein. Methods and Results: Oxidative burst (NOX2-dependent formation of ROS) was measured by L-012-based chemiluminescence and was largely absent in whole blood of NOX2 knockout mice. Protein expression was still detectable in different tissues of the NOX2 knockout mice, at the expected and a slightly lower molecular weight (determined by Western blot). The NOX2 gene was even largely enhanced at its expressional level in NOX2 knockout mice. RNA sequencing revealed a modified NOX2 mRNA in the knockout mice that is obviously translated to a truncated inactive mutant enzyme. Conclusion: Although the commercial NOX2 knockout mice display no considerable enzymatic NOX2 activity, expression of the NOX2 gene (when using standard primers) and protein (when using antibodies binding to the carboxy-terminal end) can still be detected, which may lead to confusion among investigators.

## 1. Introduction

Using oxygen for enhanced energy production is inevitably linked to the production of reactive oxygen species (ROS), such as superoxide, hydrogen peroxide, and hydroxyl radicals, which may have harmful effects on normal cellular function [1]. A tight balance between generation and detoxification of ROS has been shown to modulate cell physiology and development through redox signaling (low concentrations of ROS acting as signal molecules in physiologic processes) [2] and oxidative stress (high concentrations of ROS exceeding the detoxification ability of cells) [3]. Oxidative stress results in destruction of proteins, DNA, and membrane lipids [4] and has been described to be involved in carcinogenesis [5], cardiovascular diseases [6,7], and aging [8]. 

In innate immune reactions, neutrophils are cells essential for killing bacteria via activation of a wide variety of effector responses and generation of large amounts of ROS, also named an “oxidative burst” [9]. These huge amounts of ROS in neutrophils are mostly generated by activation of the isoform 2 of the superoxide-generating enzyme nicotinamide adenine dinucleotide phosphate (NADPH) oxidase (NOX2 or gp91^phox^) [10], which also have diagnostic implications in patients with peripheral artery disease [11]. 

The NOX enzyme family members, NOX1, NOX2, NOX3, NOX4, and NOX5 and the dual oxidase (DUOX) enzymes, DUOX1 and DUOX2, are membrane-associated hetero-oligomeric complexes that generate ROS by using oxygen and NADH/NADPH as the substrate and cofactor [12,13]. NOX enzymes are expressed in a cell-specific or tissue-specific manner and are rapidly activated in response to external stressors to generate large amounts of ROS. NOX2 or gp91^phox^ is the main catalytic subunit of the leukocyte NADPH oxidase complex. It is highly expressed in phagocytes (neutrophils, monocytes, macrophages, and dendritic cells) and at much lower levels in lymphocytes, endothelial cells, and colonic epithelial cells [3,14]. The significant contribution of NOX isoforms to the onset and progression of numerous diseases has largely stimulated the development of pharmacological inhibitors to treat these pathophysiological conditions, and several drug candidates reached the advanced phase clinical trials [15]. 

In our previous studies, we used the mouse model of inactivation of the mouse cybb gene (NOX2ko) to analyze the role of NOX2-generated ROS in cardiovascular diseases [16,17,18,19]. We clearly showed that, in NOX2ko mice, the respiratory burst in isolated white blood cells is nearly absent. However, we observed a clear expression of a NOX2ko protein detected by specific NOX2 antibodies in NOX2ko cell extracts and tissue homogenates. To solve these contrary results, we analyzed the NOX2 locus sequence and the NOX2ko mRNA expression in more detail.

## 2. Materials and Methods 

### 2.1. Materials

PMSF, leupeptin, aprotinin, agarose, monoclonal anti-β-tubulin antibodies, horseradish-peroxidase-coupled, and anti-mouse IgG were purchased from Sigma, Deisenhofen, Germany. Restriction enzymes, Taq polymerase, Klenow DNA polymerase, and deoxynucleoside triphosphates (dNTPs) were purchased from New England Biolabs, Frankfurt, Germany. All oligonucleotides were from Sigma, Deisenhofen, Germany. The High-Capacity cDNA Reverse Transcription Kit was purchased from Applied Biosystems, Darmstadt, Germany. The PrecisionPLUS 2x qPCR MasterMix with SYBR green was obtained from Primer Design, Chandler’s Ford, United Kingdom. Primer-Probe-Mixes of murine NADPH oxidase (NOX-2 Mm00432775_m1) and murine TATA box binding protein (TBP, Mm_00446973_m1) were purchased from Applied Biosystems, Foster City, CA, USA. The mouse monoclonal gp91^phox^ antibody was obtained from BD Biosciences, USA. The secondary antibody peroxidase-labeled goat-anti-mouse (GAM-POX), avidin-biotin complex (ABC) reagent, and DAB reagent (used for IHC) were from Vector Laboratories, CA, USA. The polyclonal NOX-2 antibody used for immunohistochemistry was obtained from LSBio, Seattle, WA, USA. The biotinylated secondary antibody was from Thermo Fisher Scientific, Waltham, MA, USA. The Bradford reagent mix for determination of protein concentration was obtained from BioRad, Munich, Germany.

### 2.2. Mice and Approval of Animal Studies

Wildtype (WT) C57BL/6J mice, NOX2ko, and p47phox-ko mice were obtained from Jackson Lab (B6.129S-Cybb^tm1Din/J^, stock number 002,365 for NOX2ko; B6N.129S2-Ncf1^tm1Shl^/J, stock number 027,331 for p47phox-ko) and maintained in the Translational Animal Research Center of the JGU Mainz. The NOX2ko(NOX2ko) mice were generated by insertion of a fragment containing a neomycin resistance gene (neoR) under the control of the mouse phosphoglycerate kinase 1 gene (PGK1) promoter and polyadenylation signal (polyA) into exon 3 of the gene (target exon mutant, see subsequent figures for a map [20]). These NOX2-deficient mice were referred to as NOX2ko mice. Both strains had access to water and standard chow diet ad libitum. Genotyping of the animals was performed by PCR, using the primers that are recommended by the Jackson Lab and span the region of the wildtype NOX2 gene flanking exon 3. The oligonucleotides shown in Table 1 were used for genotyping the NOX2-locus:

All animal studies were permitted by the Ethical Committee and Landesuntersuchungsamt Rheinland-Pfalz (ID 23177-07/G18-1-053, 23177-07/G15-1-094 and 23177-07/G16-1-055). 

### 2.3. Measurement of Oxidative Burst in Whole Blood

A leukocyte-dependent oxidative burst was read-out for leukocyte-dependent hydrogen peroxide formation (mainly derived from dismutation of superoxide generated by phagocyte-type NADPH oxidase, Nox2 [21]), as described [10,16,22]. Hydrogen peroxide was converted by myeloperoxidase to highly reactive oxygen-metal complexes that lead to oxidation of L-012 to an intermediate that, by chemical reaction, emits energy in the form of chemiluminescent light. Oxidative burst was measured in fresh citrate blood upon dilution 1:50 and stimulation with zymosan A (50 µg/mL), as well as phorbol ester dibutyrate (10 µM) in PBS buffer, containing Ca^2+^/Mg^2+^ (1 mM) by L-012 (100 µM) enhanced chemiluminescence (ECL) using a Mithras2 chemiluminescence plate reader (Berthold Technologies, Bad Wildbad, Germany). L-012 (8-amino-5-chloro-7-phenylpyrido [3,4-d]pyridazine-1,4-(2H,3H)dione sodium salt) was purchased from Wako Pure Chemical Industries (Osaka, Japan).

### 2.4. Analysis of NOX2ko mRNA by RT-PCR

To proof the sequence data and analyze the NOX2ko mRNA expression, qualitative RT-PCR experiments were performed as described before [23], using the oligonucleotides listed in Table 2.

The products of the RT-PCR reactions were analyzed by electrophoresis on 2% agarose gels. For comparison, a DNA marker ladder was used.

### 2.5. Analysis of NOX2ko mRNA Structure and Expression Values Using NGS Data

To proof the sequence data and analyze the NOX2ko mRNA expression in leukemia-initiating stem cells, next generation sequencing (NGS) sequence reads published by Adane et al. ([24], GEO: GSE117657) were analyzed with CLC genomics workbench (QIAGEN, version 20.4), as described by the manufacturer (using the preinstalled parameters). The reads were aligned to the NOX2ko mRNA sequence using the “Map reads to reference” algorithm with the following parameters: match score = 1, mismatch cost = 2, linear gap cost, length fraction = 0.5, similarity fraction = 0.8, map randomly. For expression analyses. The “RNA-Seq analyses” algorithm was used (using the preinstalled parameters and the Mus musculus sequences provided by the manufacturer). The NOX2ko data were compared to the NOX2wt data. To align the murine NOX2wt and NOX2ko, a protein sequence MacVector (version 17.5.4, using the preinstalled parameters) was used.

### 2.6. Real-Time Reverse Transcription Polymerase Chain Reaction Analysis

Total mRNA from the frontal cortex of the brain was isolated using the RNeasy Fibrous Tissue Mini Kit, Qiagen, Hilden, Germany, according to the manufacturers protocol. A total of 50 ng RNA was used for quantitative reverse transcription real-time PCR (qRT-PCR) analysis using QuantiTect Probe RT-PCR kit (Qiagen), as described previously [16,25]. Primer-Probe-Mixes purchased from Applied Biosystems (Foster City, CA) were used to analyze the mRNA expression patterns of NADPH oxidase (NOX-2 Mm00432775_m1, binds to exon 3 and 4 of the mRNA, see figures below) and normalized on the TATA box binding protein (TBP, Mm_00446973_m1) as an internal control. For quantification of the relative mRNA expression, the comparative ΔΔCt method was used. Gene expression of the target gene in each sample was expressed as the percentage of wildtype. mRNA expression in liver and spleen tissues was quantified in a two-step real-time RT-PCR using SYBR Green, as described before [23], with the oligonucleotides listed in Table 3. All primer/probe sets had efficiencies of 95% (±5%).

To calculate the relative expression of glyceraldehyde 3-phosphate dehydrogenase (GAPDH)-, NOX2wt-, or NOX2ko-mRNA, the 2^(‚∆∆C(T))^ method [26] was used. According to this method, the C(T) values for NOX2wt or NOX2ko-mRNA expression in each sample were normalized to the C(T) values of GAPDH-mRNA in the same sample. Then the values of samples obtained from NOX2 wt animals were set to 100%, and the percentage of NOX2wt- or NOX2ko-expression was calculated. 

### 2.7. Western Blot Experiments

The procedures were similar to those described recently [16,25]. All protein homogenates were boiled in Laemmli buffer to remove disulfide bridges and unfold the proteins and Loading and transfer of proteins was assessed by Ponceau S staining of the nitrocellulose membrane. Protein samples from different tissues (25 µg RAW 264.7 macrophages and aorta, 40 µg heart and lung) were analyzed by Western blot analysis for the NADPH oxidase subunit NOX2 (mouse monoclonal gp91^phox^, 1:1000, BD Biosciences, USA, directed against a peptide containing the amino acids from position 450 to 556 of the murine NOX2 protein) and visualized using a secondary antibody with a covalently bound peroxidase moiety (GAM-POX, 1:10,000, Vector Laboratories, CA, USA). All incubation and washing steps were performed according to the manufacturer’s instructions. Visualization of antibody-specific signals was performed using the Super Signal ECL kit from Thermo Scientific and a ChemiLux Imager (CsX-1400M, Intas, Göttingen, Germany).

### 2.8. Immunohistochemistry

The first lung segments were fixed in paraformaldehyde (4%) and paraffin-embedded. Paraffin-embedded lung samples were cut to thin sections and stained with the antibody [19]. NOX-2 was stained with a specific polyclonal antibody (LSBio, Seattle, WA, USA, #B12365, 1:200). The biotinylated secondary antibody (Thermo Fisher Scientific, Waltham, MA, USA) was used at a dilution of 1:1,100, according to the manufacturer’s instructions. For immunochemical detection, the ABC reagent (Vector) and then the DAB reagent (peroxidase substrate Kit, Vector) were used as substrates.

### 2.9. Statistics

Data represent means ± SEM. Statistical differences were determined by factorial analysis of variance, followed by “Tukey’s” or “Dunnett’s” multiple comparison test. In the case of two means, classical two-sided t-test analyses were used. All statistical analyses were performed using Graphpad Prism 8. 

## 3. Results

In our attempts to analyze the importance of ROS generated by NOX2, we used the B6.129S-Cybb^tm1Din^/J mice (also named gp91phox^−/−^, NOX2ko, or NOX2ko) offered by the Jackson Laboratory as the animal model.

**Figure 1:**
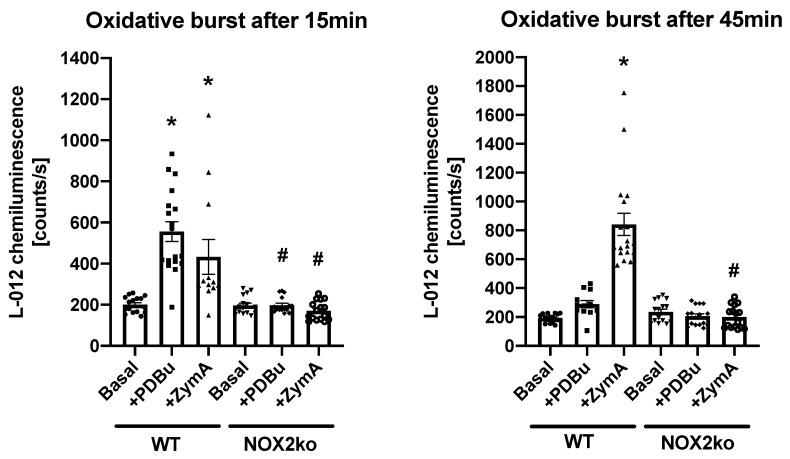
Characterization of the oxidative burst signals in whole blood from wildtype (wt, **WT**) and **NOX2ko** animals. Oxidative burst as a read-out for leukocyte NADPH oxidase activity was determined upon stimulation of whole blood (1:50 in PBS with 1 mM Ca^2+^/Mg^+^) with phorbol ester dibutyrate (PDBu, 10 µM) or zymosan A (ZymA, 50 µg/mL) by L-012- (100 µM) enhanced chemiluminescence. The oxidative burst signal was determined at 15- or 45-min post addition of the stimulus. Data are mean ± SM from *n* = 12–18 measurements with pooled mouse blood samples. The data were re-evaluated and merged from raw data in [16,19] with permission. * = *p* < 0.05 vs. WT basal; # = *p* < 0.05 vs. respective WT group with the same treatment.

Alongside performing genotyping, as recommended by the Jackson Laboratory (see Material and Methods using the primers com, wt, and mut). We also performed analyses to measure the oxidative burst in whole blood from wt and NOX2ko animals. As shown in Figure 1, we observed a nearly complete inhibition of ROS production increase in response to phorbol ester (PDBu) or zymosan A (ZymA) in whole blood from NOX2ko animals compared to wt cells.

**Figure 2:**
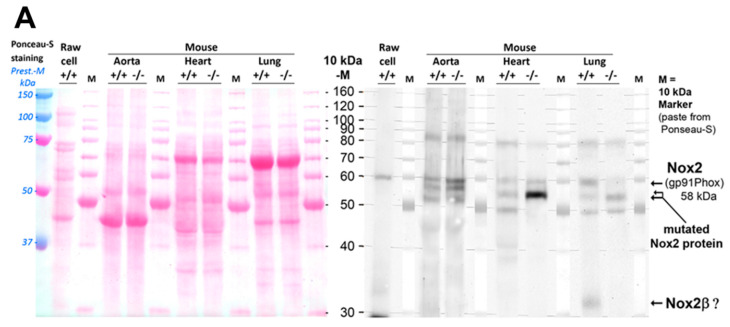

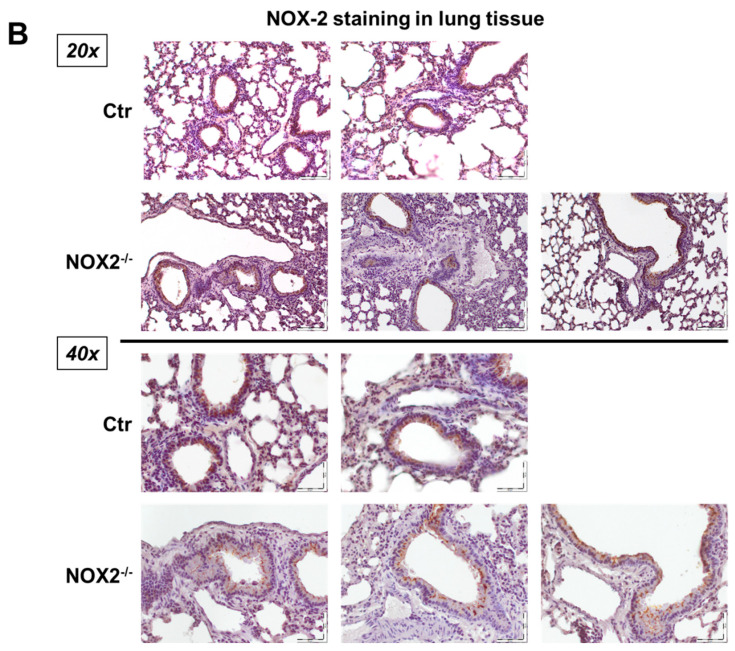
Protein expression of NOX2 in different tissues. (**A**) Western blot analysis with different tissues from wt (+/+) and NOX2ko (−/−) mice (protein/lane: 25 µg RAW cell (as the NOX2 positive control) and 25 µg aorta, 40 µg heart and lung). Signals for NOX2 were observed at 58 kDa in wildtype mice, and additionally as smaller sized bands in NOX2ko mice, especially with stronger intensity noticed in the heart. Additionally, the ≈ 30 kDa NOX2β isoform, as described by Harrison et al. [27], was probably detected in lung tissues. Loading and transfer of proteins was assessed by Ponceau S staining of the nitrocellulose membrane. (**B**) Immunohistochemical staining for pulmonary NOX2 expression. Signals for NOX2 were observed in lungs of wildtype and NOX2ko mice (brown color). The nuclei were stained by a blue color. Representative microscopy images are shown for two control (wildtype) and three NOX2ko mice. Magnification is 20× and 40×.

To ensure that this block in the oxidative burst in cells of NOX2ko animals resulted from inhibition of NOX2 expression, we performed Western blot experiments with different tissues of wt and NOX2ko animals using a NOX2-specific antibody (generated using a peptide containing the amino acids from position 450–556 of the murine NOX2 protein carboxy-terminal). Different tissues were used to ensure that our observation was not limited to a cell-specific effect. As shown in Figure 2A, there was a clear expression of a NOX2-antibody detected protein (NOX2ko-prot) in different tissues of the NOX2ko animals. Additionally, this protein displayed an enhanced expression compared to the NOX2 expression in wt tissues. However, a closer inspection of the data showed a reduction in the molecular weight NOX2ko-prot compared to the wildtype NOX2 protein. Expression of a NOX2 protein in lungs of NOX2ko animals was also confirmed by immunohistochemical staining (Figure 2B). As described by Harrison et al. [27], we detected a ≈ 30 kD protein in the lung and, potentially, RAW cell homogenates (but not in the aorta or heart), which is likely to be the NOX2β isoform. This isoform was not expressed in NOX2ko animals, supporting the data described by Harrison et al. Of note, different levels of N-glycosylation of proteins (e.g., in different tissues or species) could also generate bands at different molecular weight. As shown by Pollock et al., deglycosylation of NOX2 using N-glycoside F resulted in a band running at a lower molecular weight [20]. Although most N-glycosylation should have been removed in our samples due to the boiling procedure in Laemmli buffer, we cannot exclude that the deglycosylation process was incomplete, resulting in varying molecular weight bands, as we did not use enzymatic deglycosylation for validation. It is important that we observe immunoreactivity for NOX2 in the knockout mice using two different methods (Western blot and immunohistochemistry) that mostly support the results presented below on gene expression, although the final proof of the truncated protein band by a highly specific antibody or blocking peptide remains to be provided.

**Figure 3:**
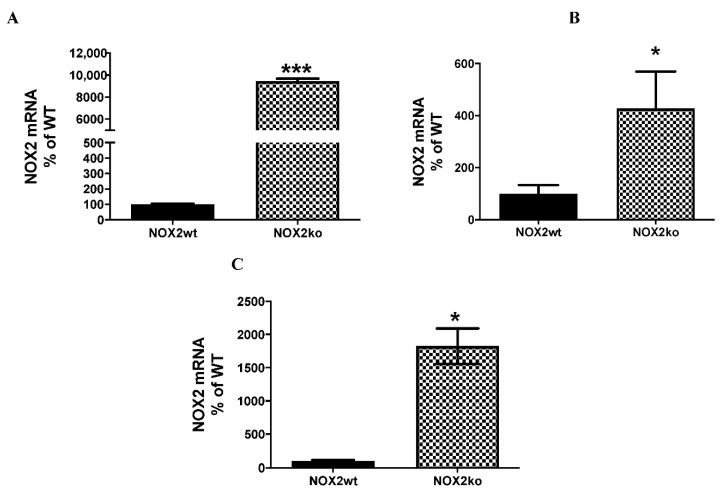
Analysis of the NOX2 mRNA expression by qRT-PCR (**A**,**B**) or NGS-analyses (**C**). (**A**) Shown is the statistical analysis of qRT-PCR experiments performed with RNA isolated from the brain of NOX2 wt (NOX2wt) or NOX2ko (NOX2ko) animals using total brain homogenates and the primer-probe-mixes purchased from Applied Biosystems. The relative NOX2 mRNA expression in NOX2wt animals was set to 100%. Shown are the mean ± SEM of *n* = 6–8 experiments (*** *p* < 0.001 vs. NOX2wt; two tailed unpaired *t*-test). (**B**) Shown is the statistical analysis of qRT-PCR experiments performed with RNA isolated from the brain of NOX2 wt (NOX2wt) or NOX2ko (NOX2ko) animals using total liver homogenates and the primers qRT-PCR-5P2/3P2 shown in the Materials and Methods section. The relative NOX2 mRNA expression in NOX2wt animals was set to 100%. Shown are the mean ± SEM of *n* = 8 experiments (* *p* < 0.05 vs. NOX2wt; two tailed unpaired *t*-test). (**C**) Shown is the statistical analysis of the mRNA expression analyzed using the NGS data published by Adane et al. ([24], GEO: GSE117657). The RNAs were isolated from leukemia-initiating stem cells. The rpkm (reads per kilo base per million mapped reads) data of the NOX2 wt (NOX2wt) animals were set to 100%. Shown are the mean ± SEM of *n* = 2 experiments (* *p* < 0.05 vs. NOX2wt; two tailed unpaired *t*-test).

**Figure 4:**
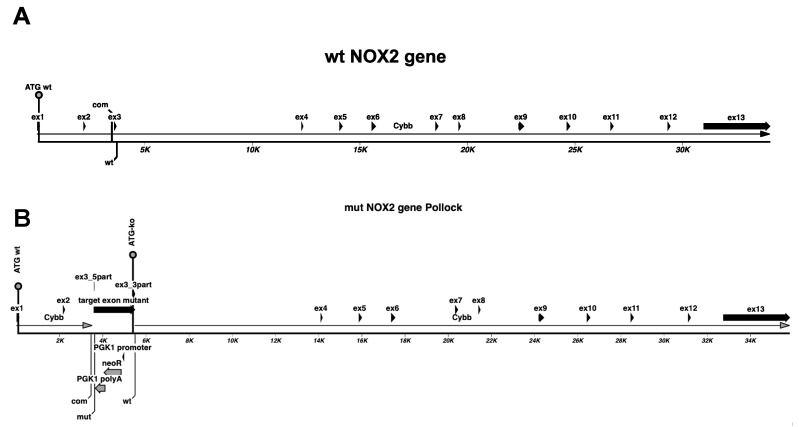
Structure of the murine wildtype NOX2 gene (**A**) and the inactivated NOX2 gene (**B**) in NOX2ko mice. (**A**) Shown is a schematic picture of the murine wildtype NOX2 (gp91phox) gene (mouse Chromosome X, NC_000086.7 (9,435,252.9487766, complement)). (**B**) Shown is a schematic picture of the inactivated NOX2 gene (as describe by [20]). ATG = translational start codon; Cybb = cybb/NOX2/gp91phox gene sequence; ex = exon; ko = inactivated gene, knockout; neoR = neomycin resistance gene; PGK1polyA = mouse phosphoglycerate kinase 1 gene polyadenylation signal; PGK1 promoter = mouse phosphoglycerate kinase 1 gene promoter sequence; wt = wildtype.

**Figure 5:**
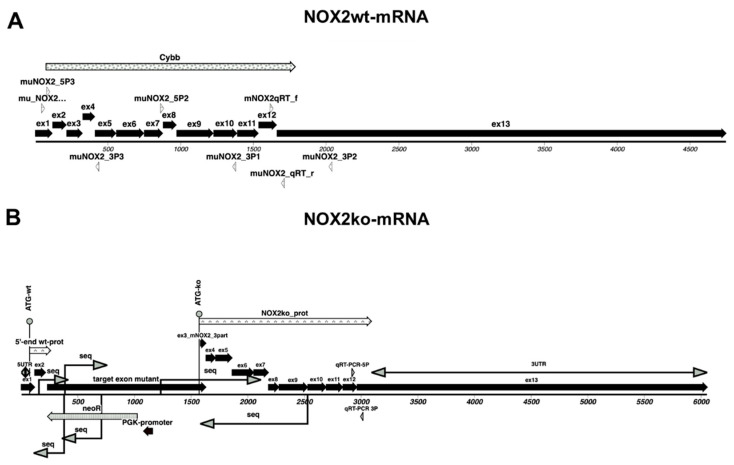
Structure of the mRNAs encoded by the murine wt NOX2 gene (**A**) and the inactivated NOX2 gene (**B**) in NOX2ko mice. (**A**) Shown is a schematic picture of the mRNA encoded by the murine wildtype NOX2 (gp91phox) gene (NM_007807). (**B**) Shown is a schematic picture of the mRNA encoded by the inactivated NOX2 gene. 5′-end wt-prot = sequence of the putative small 5′-part of the wt NOX2 protein; ATG = translational start codon; ex = exon; Mm00432775_m1 = location of the PCRF generated in the qRT-PCR assays using RNA isolated from brain tissues; neoR = neomycin resistance gene; NOX2ko_f and NOX2ko_r = primer used for qualitative RT-PCR analyses detecting the mutated NOX2 mRNA only; NOX2ko_prot = sequence of the fusion protein encoded by the mutated NOX2 mRNA; NOX2wt_f and NOX2wt_r = primer used for qualitative qRT-PCR analyses, detecting both the wt and mutated NOX2 mRNA; PGK1 promoter = mouse phosphoglycerate kinase gene promoter sequence; qRT-PCR-5P2 and -3P2 = primers used for quantitative RT-PCR with RNA isolated from liver tissues; seq = sequenced part of the mRNA; UTR = untranslated region; wt = wildtype.

**Figure 6:**
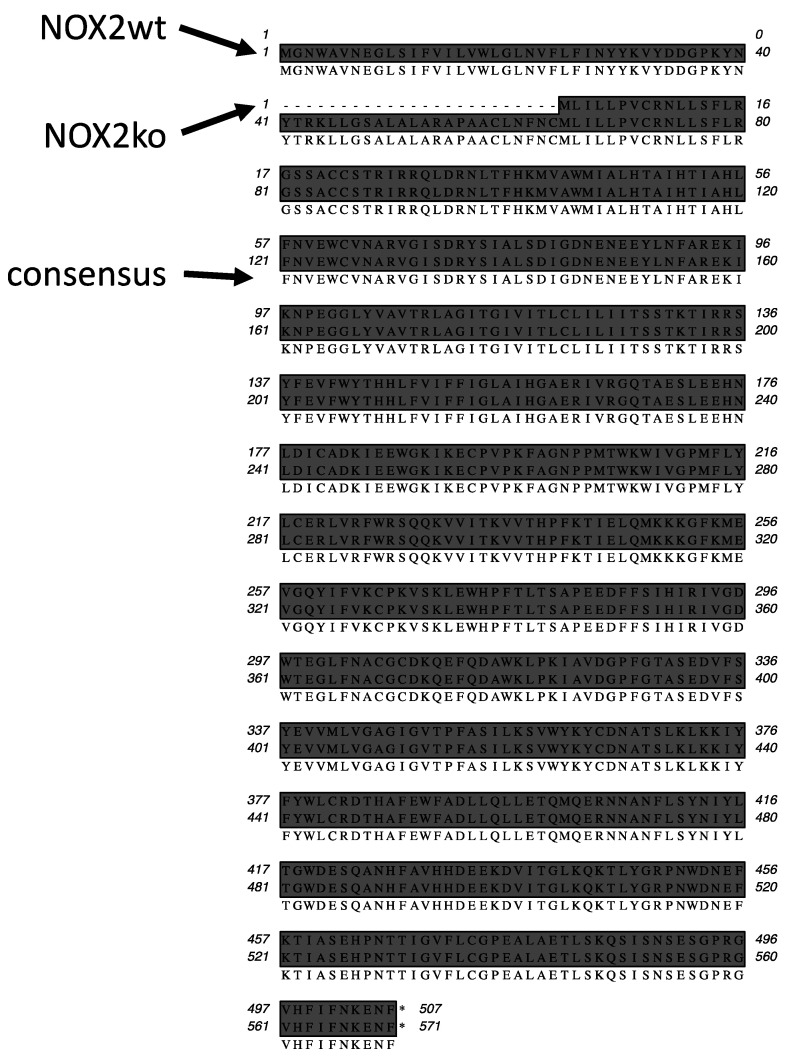
Alignment of sequences of the proteins encoded by the NOX2wt and NOX2ko mRNA. Additionally, the consensus amino acid sequence is shown. Shown is a picture of the alignment of the wt and mutated NOX2 mRNAs. The wt NOX2 mRNA encodes a protein with 571 amino acids, whereas the NOX2ko mRNA codes for a 507 amino acid protein (uppermost sequence = NOX2ko; middle sequence = wt; lowest sequence = consensus).

To gain further insight into this surprising result of the elevated expression of a NOX2ko protein in NOX2ko animals, we also analyzed the NOX2 mRNA expression in the brains and liver of NOX2ko animals by qRT-PCR, using primer systems described in the Materials and Methods section (location of the primer binding in Figure 3, Figure 4, Figure 5 and Figure 6 below). As shown in Figure 3A,B, we observed a marked enhancement of the NOX2 mRNA expression in the NOX2ko brains and livers compared to wt animals. Using the RNA seq data of Adane et al. [24] (GEO: GSE117657) to quantify the NOX2 mRNA expression in leukemia-initiating stem cells, we also observed a marked enhancement of the NOX2 mRNA expression in NOX2ko animals (see Figure 3C). 

To clarify these contrary results, we performed a deeper analysis of the DNA sequence of the NOX2 locus in NOX2ko mice. As described by Pollock et al. [20], the NOX2ko mice were generated by insertion of a neomycin resistance gene (neoR) under the control of the murine phosphoglycerate kinase 1 (PGK1) promoter and poly adenylation (polyA) signal into exon 3 of the murine NOX2 gene (see Figure 4). As shown in Figure 5, transcription of the mutated NOX2 gene in NOX2ko cells should result in a NOX2ko-mRNA encoding a NOX2ko protein (NOX2ko-prot), which contains the 506 carboxy terminal amino acids of the NOX2wt protein (see Figure 6 for an alignment of the amino acid sequences).

**Figure 7:**
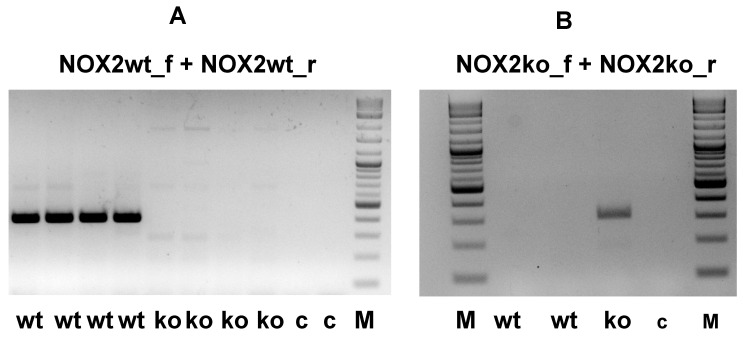
Qualitative RT-PCR reaction using RNAs isolated from liver from NOX2wt and NOX2ko animals. (**A**) Shown is a representative picture of an agarose gel analyzing the RT-PCR products using RNAs isolated from the liver of NOX2 wildtype (wt) and NOX2ko (ko) animals. The RT reaction was performed with oligo-dT primers. The PCR reaction was performed with the primers NOX2wt_f and NOX2wt_r (see Figure 5B for the location of the primers). This primer pair resulted in a 395 bp DNA fragment only in PCR reactions using RNA from wt animals. (**B**) Shown is a representative picture of an agarose gel analyzing the RT-PCR products using RNAs isolated from the liver of NOX2 wildtype (wt) and NOX2ko (ko) animals. The RT reaction was performed with oligo-dT primers. The PCR reaction was performed with the primers NOX2ko_f and NOX2ko_r (see Figure 5B for the location of the primers). This primer pair resulted in a 306 bp DNA fragment only in PCR reactions using RNA from ko animals. c = control reaction without RNA; ko = RNA from NOX2 ko animals; M = DNA marker; wt = wildtype.

**Figure 8:**
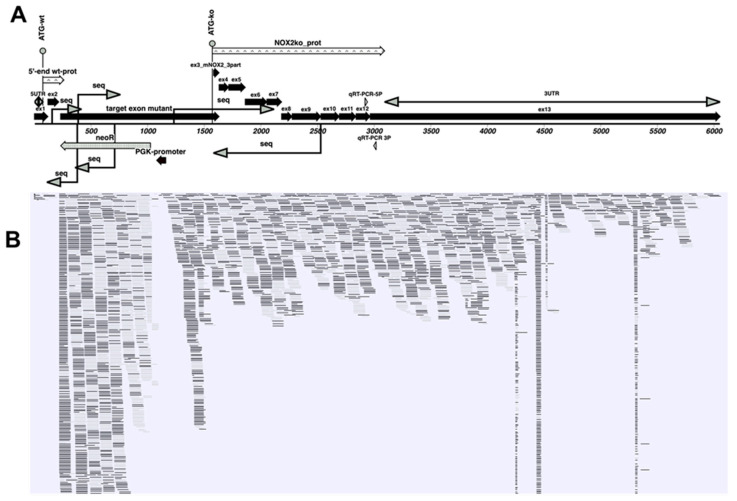
Alignment of NGS reads to the mRNA expressed in NOX2ko animals. (**A**) Shown is a schematic picture of the mRNA encoded by the inactivated NOX2 gene (as described by [20], which is the same as Figure 5B). (**B**) Shown is a picture of the alignment of NGS reads, obtained from RNA, isolated from leukemia-initiating stem cells, obtained from NOX2ko animals ([24], GEO: GSE117657) to the mRNA sequence of the mutated NOX2 gene in NOX2ko animals. The NGS sequence reads published by Adane et al. [24] (GEO: GSE117657) were analyzed with a CLC genomics workbench (QIAGEN, version 20.0.4), as described by the manufacturer (using the preinstalled parameters). The reads were aligned to the NOX2ko mRNA sequence using the “Map reads to reference” algorithm. Shown are reads with >99% identity. The black rectangle are reads of the upper strand, whereas the gray rectangles are reverse reads (lower strand). 5′-end wt-prot = sequence of the putative small 5′-part of the wt NOX2 protein; ATG = translational start codon; ex = exon; Mm00432775_m1 = location of the PCR fragment generated in the qRT-PCR assays using RNA isolated from brain tissues; neoR = neomycin resistance gene; NOX2ko_f and NOX2ko_r = primer used for qualitative RT-PCR analyses detecting the mutated NOX2 mRNA only; NOX2ko_prot = sequence of the fusion protein encoded by the mutated NOX2 mRNA; NOX2wt_f and NOX2wt_r = primer used for qualitative qRT-PCR analyses, detecting both the wt and mutated NOX2 mRNA; PGK1 promoter = mouse phosphoglycerate kinase gene promoter sequence; qRT-PCR-5P2 and -3P2 = primers used for quantitative RT-PCR with RNA isolated from liver tissues; seq = sequenced part of the mRNA; UTR = untranslated region; wt = wildtype.

To prove the expression of this NOX2ko mRNA, we performed qualitative RT-PCR experiments using liver tissues of NOX2wt and NOX2ko animals using the primers NOX2wt_f and NOX2wt_r (detects only the wt mRNA), as well as NOX2ko_f and NOX2_ko_r (detects only the ko mRNA; locations are indicated in Figure 5). As shown in Figure 7, performing qRT-PCR with RNA isolated from liver tissues of different NOX2wt and -ko animals, we detected the corresponding bands (wt 395 bp, ko 306 bp) using the wt primer pair in wt probes only and the ko primer pair in ko probes only. Additionally, we performed sequencing of different parts of the NOX2ko mRNA (shown as seq in Figure 5B). Our sequence data fit very well with the deduced NOX2ko mRNA sequence. In addition, we used the NGS reads published by Adane et al. [24] (GEO: GSE117657) to perform alignments of the sequence reads to the NOX2ko mRNA sequence. As shown in Figure 8, these analyses resulted in nearly 100% alignment of the NOX2ko mRNA sequence to different sequence reads. 

**Figure 9:**
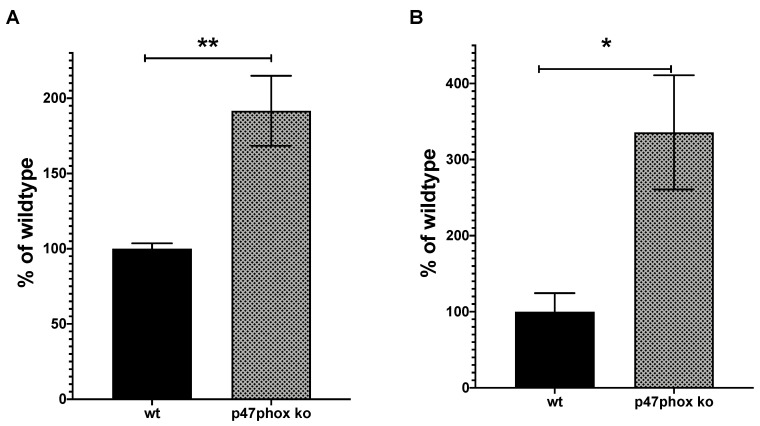
Analysis of the NOX2 mRNA expression in mice with the inactivated p47phox gene by qRT-PCR. (**A**) Shown is the statistical analysis of qRT-PCR experiments performed with RNA isolated from the liver of NOX2 wt or animals with the inactivated p47phox gene (p47phox ko) using total liver homogenates and the primers qRT-PCR-5P2/3P2 shown in the Materials and Methods section. The relative NOX2 mRNA expression in NOX2wt animals was set to 100%. Shown are the mean ± SEM of *n* = 6–8 experiments (** *p* < 0.01 vs. NOX2wt; two-sided unpaired *t*-test). (**B**) Shown is the statistical analysis of qRT-PCR experiments performed with RNA isolated from the spleen of NOX2 wt (NOX2wt) or animals with inactivated p47phox gene (p47phox ko), using total spleen homogenates and the primers qRT-PCR-5P2/3P2 shown in the Materials and Methods section. The relative NOX2 mRNA expression in NOX2wt animals was set to 100%. Shown are the mean ± SEM of *n* = 6 experiments (* *p* < 0.05 vs. NOX2wt; two tailed unpaired *t*-test).

To get a hint to the mechanisms involved in the enhanced expression of the mutant NOX2 mRNA as described above (see Figure 3), we analyzed the expression of the NOX2 mRNA in mice with the inactivated p47phox gene (another subunit of the NOX2 complex), also leading to NOX2 complex inactivation [28]. As shown in Figure 9, inactivation of the NOX2 complex by genetic deficiency of the p47phox subunit resulted in significant enhancement of the endogenous NOX2 mRNA expression in the liver and spleen of the p47phox-ko animals.

## 4. Discussion

With the present study, we demonstrated that the NOX2ko mice (obtained from Jackson Lab, B6.129S-Cybbtm1Din/J, stock number 002365) expressed an inactive form of the NOX2 protein. The knockout mice showed NOX2 protein signals in different tissues, as assessed by Western blot analysis and immunohistochemical staining. NOX2 gene expression was even upregulated 6- to 90-fold (NGS or qRT-PCR data) in brain, liver, or leukemia-initiating stem cells obtained from NOX2ko animals. In sharp contrast, whole blood from NOX2ko animals showed no oxidative burst in response to potent activators of NOX2, such as phorbol ester or zymosan A [10], clearly indicating that the leukocytes in the whole blood of NOX2ko mice lacked enzymatic NOX2 activity. We can now explain these findings by complete sequencing of the modified NOX2 mRNA in the knockout mouse, which is translated to a truncated inactive mutant enzyme that can still be detected by certain antibodies that bind to the carboxy-terminal part of the protein.

Genetic Nox2 deletion has been used for almost 2 decades to study the impact of NOX2-derived ROS on disease development and progression. A critical role of the NADPH oxidase isoform NOX2 (gp91phox) was demonstrated for angiotensin II (AT-II) induced hypertension by using NOX2ko or overexpressing NOX2*tg* mice [29,30,31,32], which was further refined by providing the molecular proof that NOX2 in immune cells (LysM-positive myelomonocytic cells) is key to the development of hypertension in angiotensin-II infused mice [33]. Likewise, the essential role of NOX2 was proven for ischemia/reperfusion damage in experimental myocardial infarction [34,35] or for the progression of diabetes [36]. Despite ample evidence for a central role of NOX2 in numerous disease settings coming from these animal models, the translation of these results to the clinical situations is not easy. Reasons for this are insufficient specificity of pharmacological inhibitors for the different NOX isoforms, compound toxicity [37,38], and, finally, the physiological roles of NOX enzymes in central cellular processes, such as differentiation, proliferation, and migration (mostly NOX4) [39,40,41,42] thyroid hormone formation (mostly DUOX) [43], as well as immune processes and host defense (mostly NOX2 and potentially NOX1) [44,45,46]. 

The essential role of NADPH oxidase in host antimicrobial responses is shown by the chronic granulomatous disease (CGD) [46]. The most common and severe form of CGD, X-linked CGD, is caused by a mutation in the catalytic gp91^phox^ subunit of the NOX2 complex; however, mutations in other NOX2 subunits can also cause CGD [47,48,49]. CGD patients suffer from life-threatening bacterial and fungal infections, resulting in high mortality and morbidity. Over-representation of certain bacterial infections caused by catalase positive microorganisms, such as *Staphylococcus aureus, Pseudomonas*, and *Burkholderia cepacia*, as well as fungal species, such as *Aspergillus*, are common. Thus, the oxidants generated by NADPH oxidase are critical mediators of host anti-microbial defense. Alongside these considerations, the lack of specificity for the different NOX isoforms may represent a major reason for why, so far, NADPH oxidase inhibitors are not used in clinics [38,50].

In addition, data from CGD patients, as well as mouse models [20], demonstrate profound dysregulation of host inflammatory responses, neutrophil hyperactivation, and tissue damage in response to microbial ligands or tissue trauma. There is evidence for a critical role for NOX2-derived ROS in the modulation of neutrophil responses during inflammation [9]. In addition to their anti-microbial function, NOX2-produced ROS modulate multiple cellular processes, such as antigen presentation in B cells [51], T cell receptor stimulation [52], and subset polarization [53,54]. NOX2-generated ROS function as essential signaling molecules that are essential for the regulation of neutrophil responses during priming, degranulation, neutrophil extracellular trap formation, and stimulation of central regulators of inflammatory responses, such as NLRP3 inflammasome and HMGB1 protein, as well as apoptosis, independent of their role in microbial killing [6,9]. This may be another reason for why NOX2 inhibitors show serious side effects.

The enhancement of the expression of the NOX2ko mRNA in comparison to the NOX2wt mRNA, as seen in brain, liver, and leukemia-initiating stem cells [24], may be based on different mechanisms. First, as seen, for example, by the insertional activation of oncogenes in gene therapy, approaches to cure the X-linked severe combined immunodeficiency disease (X-SCID), the strong promoter of the long terminal repeats (LTRs) of the retroviral constructs, activates neighboring promoters (in the case of X-SCID the oncogene LMO2 promoter, resulting in leukemia [55]). This insertional mutagenesis has also been used for the detection of pre-oncogenes [56]. Similar to this published mechanism, the PGK1-promoter-neoR-PGK1-polyA cassette inserted in the exon 3 of the cybb/NOX2 gene may also enhance the DNA accessibility of the NOX2 promoter and thereby enhance transcription of the mutated NOX2 locus in NOX2ko animals.

The second explanation of the enhanced NOX2ko mRNA expression may relate to the reduced ROS production in the NOX2ko animals. Our data shown in mice with the inactivated p47phox gene, and therefore the inactivated NOX2 complex, hint this explanation. The reduced ROS production in the p47phox animals lead to an enhanced expression of the endogenous NOX2 mRNA (Figure 9). As shown above, NOX2 has been described to be an important immunomodulator, also repressing the activity of the immune system (enhanced inflammation reactions in NOX2ko animals and CGD patients). Therefore, ROS produced by the NOX2 complex seems to have a very important role in the regulation of the immune system [6,57,58], which makes pharmacological targeting of NOX2 and other NADPH oxidase isoforms challenging [59]. Any therapy trying to reduce NOX2-related ROS production may have serious side effects due to this fact, as can be envisaged in patients with CGD [46]. A crucial transcription factor regulating immune gene expression is the nuclear factor κ-light-chain-enhancer of activated B cells (NF-κB) [60,61,62]. There are several links between ROS production and regulation of NF-kB activity [63,64,65]. Interestingly, ROS may activate or inhibit NF-kB activation and the expression of NF-kB-regulated genes in a context-dependent manner [66]. In alveolar type II, lung epithelial A549 cells TNF-α or IL-1β incubation result in a clear induction of NF-kB activity. Incubation of the cells with oxidants (e.g., H_2_O_2_ or ONOO^−^) after TNF-α or IL-1β exposure induced a robust and long-lasting hyperactivation of NF-κB (by inhibiting the re-synthesis of I-κB). However, preincubation of the cells with the same oxidants before TNF-α or IL-1β exposure leads to a nearly complete inhibition of NF-κB activation [66]. In addition, there are now numerous reports regarding the regulation of NF-κB by other signal transduction mechanisms [62], which have also been described to be at least partially regulated by ROS. In human phagocyte cells, NOX2 expression has been shown to be NF-κB-regulated by one NF-κB binding site in the 5′ upstream sequence to the NOX2 core promoter [67].

## 5. Conclusions

Based on our present data, it seems quite likely that inactivation of the NOX2 gene in NOX2ko animals resulted in a reduced inhibition of the NOX2 gene transcription, resulting in enhanced expression of NOX2ko mRNA in these animals.

## Figures and Tables

**Table 1:** Sequences of oligonucleotides for genotyping the NOX2-locus.

Oligonucleotide	Sequence
com	5-AAGAGAAACTCCTCTGCTGTGAA-3
wt	5-CGCACTGGAACCCCTGAGAAAGG-3
mut	5-GTTCTAATTCCATCAGAAGCTTATCG-3

**Table 2:** Sequences of oligonucleotides for qualitative RT-PCR of NOX2wt and NOX2ko mRNA.

Oligonucleotide	Sequence
**NOX2wt**	
NOX2wt_f	5-GAGGCAGAACCAACACTTAACC-3
NOX2wt_r	5-TGAAGAGATGTGCAATTGTGTG-3
**NOX2ko**	
NOX2ko_f	5-TGTCATTCTGGTGTGGTTGG-3
NOX2ko_r	5-TCTGGATTCATCGACTGTGG-3

**Table 3:** mRNA expression in liver.

Oligonucleotide.	Sequence
**NOX2**	
qRT-PCR_5P2	5-CCAACTGGGATAACGAGTTCA-3
qRT-PCR_3P2	5-GAGAGTTTCAGCCAAGGCTTC-3
**GAPDH**	
qRT-PCR_5P	5-TTCACCACCATGGAGAAGGC-3
qRT-PCR_3P	5-GGCATGGACTGTGGTCATGA-3

## Abbreviations

3′- or 5′-UTR	3′- or 5′-untranslated region
cds	coding sequence
ATG	translational start codon
Cybb	cybb/NOX2/gp9phox gene sequence
Ex	exon
neoR	neomycin resistance gene
PGK1	phosphoglycerate kinase 1
NOX2ko	mice with mutated NOX2 gene
NOX2wt	wildtype animals
PDBu	Phorbol 12,13-Dibutyrate
polyA	polyadenylation signal
qRT-PCR	quantitative real time reverse transcription polymerase chain reaction
WB	Western blot
ZymA	Zymosan A
